# Reduced mtDNA copy number increases the sensitivity of tumor cells to chemotherapeutic drugs

**DOI:** 10.1038/cddis.2015.78

**Published:** 2015-04-02

**Authors:** H Mei, S Sun, Y Bai, Y Chen, R Chai, H Li

**Affiliations:** 1 Department of Otorhinolaryngology, Research Center, Key Laboratory of Hearing Science, Ministry of Health, Affiliated Eye and ENT Hospital, Fudan University, Shanghai 200031, China; 2 Department of Otolaryngology, Children's Hospital, Chongqing Medical University, Chongqing 400014, China; 3 Co-innovation Center of Neuroregeneration, Key Laboratory for Developmental Genes and Human Disease, Ministry of Education, Institute of Life Sciences, Southeast University, Nanjing 210096, China

## Abstract

Many cancer drugs are toxic to cells by activating apoptotic pathways. Previous studies have shown that mitochondria have key roles in apoptosis in mammalian cells, but the role of mitochondrial DNA (mtDNA) copy number variation in the pathogenesis of tumor cell apoptosis remains largely unknown. We used the HEp-2, HNE2, and A549 tumor cell lines to explore the relationship between mtDNA copy number variation and cell apoptosis. We first induced apoptosis in three tumor cell lines and one normal adult human skin fibroblast cell line (HSF) with cisplatin (DDP) or doxorubicin (DOX) treatment and found that the mtDNA copy number significantly increased in apoptotic tumor cells, but not in HSF cells. We then downregulated the mtDNA copy number by transfection with shRNA-TFAM plasmids or treatment with ethidium bromide and found that the sensitivity of tumor cells to DDP or DOX was significantly increased. Furthermore, we observed that levels of reactive oxygen species (ROS) increased significantly in tumor cells with lower mtDNA copy numbers, and this might be related to a low level of antioxidant gene expression. Finally, we rescued the increase of ROS in tumor cells with lipoic acid or N-acetyl-L-cysteine and found that the apoptosis rate decreased. Our studies suggest that the increase of mtDNA copy number is a self-protective mechanism of tumor cells to prevent apoptosis and that reduced mtDNA copy number increases ROS levels in tumor cells, increases the tumor cells' sensitivity to chemotherapeutic drugs, and increases the rate of apoptosis. This research provides evidence that mtDNA copy number variation might be a promising new therapeutic target for the clinical treatment of tumors.

Mitochondria are the main site of intracellular oxidative phosphorylation and adenosine triphosphate (ATP) synthesis. Mitochondria are also involved in multiple cellular processes such as cell differentiation, cell communication and cell apoptosis. Mitochondria have their own genetic material–mitochondrial DNA (mtDNA) – that encodes 13 proteins, 22 tRNAs, and 2 rRNAs that are involved in maintaining mitochondrial function. The synthesis and degradation of mtDNA is rapid and independent of the cell cycle.^1, 2^ The dynamic equilibrium between mtDNA synthesis and degradation determines the mtDNA copy number, which can range from 10^3^ copies to 10^4^ copies in different cells.^3^

The regulation of intracellular mtDNA copy number is complicated and precise, but the exact mechanism behind this regulation remains unclear. Clay Montier *et al.*,^4^ put forward the theory of the upper and lower thresholds in which mtDNA replication switches on when the mtDNA copy number is less than the lower threshold, and mtDNA degradation starts when the mtDNA copy number is higher than the upper threshold. Mitochondrial transcription factor A (TFAM) is an important regulatory factor of mtDNA copy number. Mice that overexpress TFAM have high mtDNA copy numbers in the heart, kidney, and skeletal muscle,^5^ and knockdown of TFAM expression decreases mtDNA copy number.^6^ The phosphorylation of TFAM appears to determine its activity and, therefore, might be involved in the regulation of mtDNA copy number.^7^

Cellular mtDNA copy number is relatively stable under normal physiological conditions, and the changes in mtDNA copy number can cause pathological changes in tissues and organs. More importantly, mtDNA copy number variation has been shown to be associated with tumor development.^8, 9^ These variations cannot be explained simply by the abnormal proliferation of cells, which has significant tissue specificity. Transgenic mice with mtDNA deletions cannot survive the embryonic period, and a large number of apoptotic cells are found in these embryos.^10, 11^ In tissue-specific mtDNA knockout mice, a large number of apoptotic cells are found in the myocardium.^11^ This suggests that mtDNA copy number and apoptosis are related.

Although considerable progress has been made in understanding the molecular mechanisms of apoptosis, the exact mechanisms of tumor cell apoptosis are still not fully understood. Thus exploring the relationship between mtDNA copy number and tumor cell apoptosis can provide novel insights into these mechanisms and has significant research value.

In the current work, we induced apoptosis in HEp-2, HNE2, and A549 cells by exposing them to cisplatin (DDP) or doxorubicin (DOX) and found the mtDNA copy number significantly increased in apoptotic tumor cells. We then downregulated the mtDNA copy number to determine the impact on apoptosis and related mechanisms.

## Results

### mtDNA copy number increases in apoptotic tumor cells

**Figure 1:**
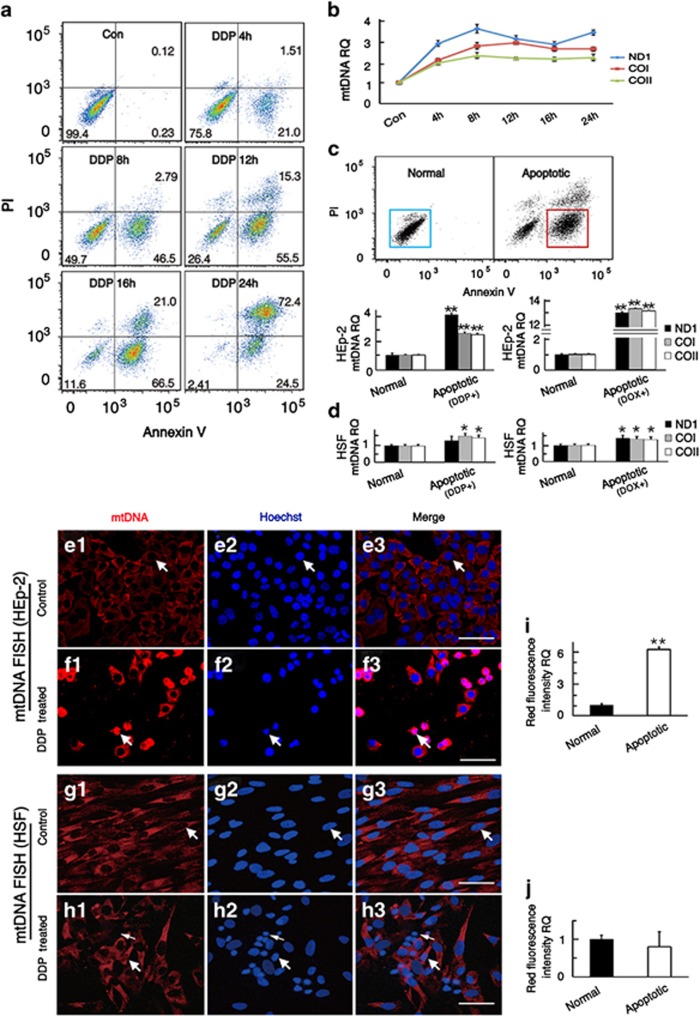
The mtDNA copy number of HEp-2 cells increased during chemotherapeutic-induced apoptosis. (**a**) Apoptosis analysis by flow cytometry. The lower left quadrant, lower right quadrant, and upper right quadrant of the images represent live cells, early apoptotic cells, and late apoptotic cells, respectively. (**b**) The cells were collected at different time points and the mtDNA copy number was analyzed by qPCR. The mtDNA genes ND1, COI, and COII were used to represent mtDNA copy number. (**c** and **d**) The results of cell sorting and qPCR. The mtDNA copy number of early apoptotic HEp-2 cells (DDP or DOX induced) was significantly higher than that in normal cells, whereas only a slight increase was observed in HSF cells. (**e**) FISH of mtDNA in the control HEp-2 cells in which the intensity of red fluorescence is indicative of mtDNA copy number. (**f**) The mtDNA copy number of apoptotic HEp-2 cells is significantly higher than control cells. (**g**) FISH of mtDNA in the control HSF cells. (**h**) The mtDNA copy number of relatively early apoptotic HSF cells was slightly increased (large white arrows), but it decreased in late apoptotic cells (small white arrows). (**i**) Quantitative analysis of red fluorescence intensity in control and apoptotic cells in **e** and **f**. (**j**) Quantitative analysis of red fluorescence intensity of control and apoptotic cells in **g** and **h**. For all experiments, the values for the normal controls were set to 1. Scale bars=20 *μ*m, *P<0.05, **P<0.01

In this experiment, flow cytometry was used to measure apoptosis and qPCR was used to determine the mtDNA copy number variation in apoptotic tumor cells after DDP or DOX treatment. We first used HEp-2 cells and found that the proportion of apoptotic cells increased gradually and peaked at 24 h after the beginning of the DDP treatment (Figure 1a). qPCR results of mtDNA copy number showed that mtDNA copy number increased and peaked at 8 h after DPP treatment and maintained high levels from 8 h to 24 h (Figure 1b). To avoid inaccuracy in determining the mtDNA copy number due to the cleavage of nuclear DNA in the later apoptotic cells, early apoptotic and normal HEp-2 cells were sorted by flow cytometry based on Annexin V-FITC/PI staining (early apoptotic cells are Annexin V-FITC–positive only) after DDP treatment for 12 h and measured by qPCR. The qPCR results based on three mtDNA genes showed that the mtDNA copy number of early apoptotic cells was significantly higher than that of normal HEp-2 cells (Figure 1c, the lower left; *t*=15.007, 13.999, and 13.291 for ND1, COI, and COII, respectively, *P*<0.01). The same results were obtained after apoptosis was induced for 24 h in HEp-2 cells by DOX (Figure 1c, the lower right). To confirm our findings, we used another two cancer cell lines—HNE2 and A549 cells—to investigate the mtDNA copy number variation after DDP or DOX treatment. Consistent with the HEp-2 cells, qPCR data showed that the mtDNA copy number of early apoptotic tumor cells was also significantly higher than normal cells (Supplementary Figures S1A and D). However, the qPCR results of HSF cells showed that the mtDNA copy number of early apoptotic cells was only slightly higher than that of normal cells (Figure 1d).

To further confirm these results, we performed separate FISH experiments. The fluorescent probe was specific for mtDNA, and thus the fluorescence intensity reflected the mtDNA copy number. In this experiment, we treated HEp-2 and HSF cells with DDP for 12 h, and the cells cultured in normal medium were used as controls. We found that the fluorescence intensity of apoptotic HEp-2 cells in the experimental group was significantly stronger than that in the control group (Figures 1e and f, i; *t*=19.014, *P*<0.01). This difference was not seen in HSF cells, and only a slight increase was seen in relatively early apoptotic cells (Figures 1g, h and j). This suggests that the mtDNA copy number is significantly increased in apoptotic HEp-2 cells but not in apoptotic HSF cells.

### Downregulation of mtDNA copy number by shRNA-TFAM sensitizes tumor cells to chemotherapeutics

**Figure 2:**
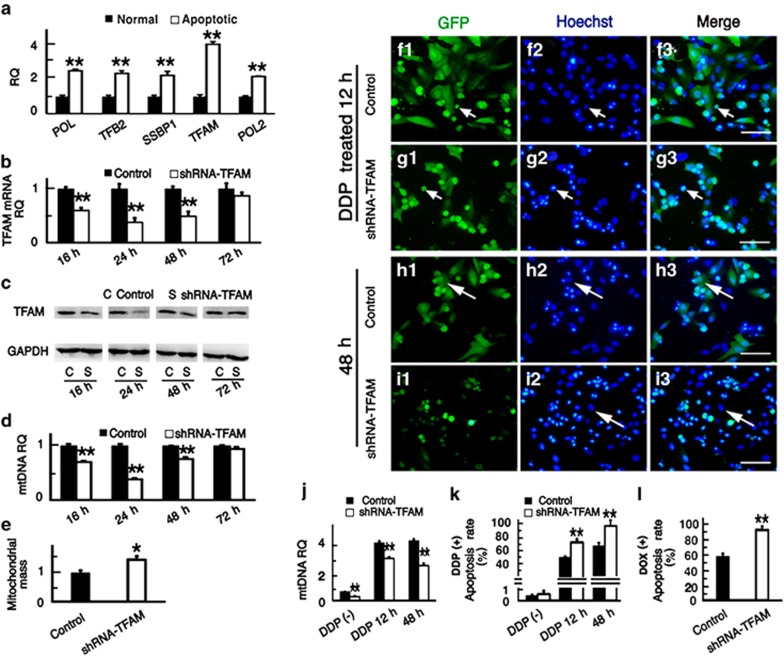
Downregulation of TFAM decreased the mtDNA copy number in HEp-2 cells and enhanced the cell sensitivity to chemotherapeutics. (**a**) The qPCR results showed that the mRNA level of TFAM was the highest among the five genes related to mtDNA copy number regulation. (**b** and **c**) The mRNA and protein levels of TFAM decreased significantly after transfection with shRNA-TFAM, reached their lowest point at 24 h after transfection, and then recovered gradually. (**d**) After transfection with shRNA-TFAM, the mtDNA copy number of HEp-2 cells decreased and reached its lowest point at 24 h. (**e**) The mitochondrial mass (mitochondria were isolated and labeled with MitoTracker Green FM) increased in cells transfected with shRNA-TFAM for 24 h. (**f**–**i**) Representative microscope images of DDP-treated cells transfected with shRNA-TFAM or control plasmids. The arrows indicate the same cell in each group (apoptotic cells, small white arrows; live cells, large white arrows). (**f**–**g**) The cells were treated with DDP for 12 h. (**h**–**i**) After treatment with DDP for 12 h, the DDP was washed away and the cells were observed at 48 h. (**j**) After plasmid transfection, the cells with GFP were sorted by flow cytometry in both the groups, and the mtDNA copy number of HEp-2 cells transfected with shRNA-TFAM was significantly decreased at the two time points after DDP treatment. (**k**) Flow cytometry data further confirmed that the apoptosis rate of cells transfected with shRNA was higher than controls at the two time points after DDP treatment. (**l**) The apoptosis rate of HEp-2 cells transfected with shRNA was also higher than controls after DOX treatment for 12 h. For all experiments, the values for the normal controls were set to 1. Scale bars=100 *μ*m, *P<0.05, **P<0.01

Early apoptotic HEp-2 cells were sorted by flow cytometry after treated with DDP for 12 h and used for microarray screening (data not shown). The expressions of five genes related to the regulation of mtDNA copy number were significantly increased in apoptotic HEp-2 cells, and these were subsequently verified by qPCR. These results showed that the expression of TFAM was increased the most (Figure 2a), and this is in line with previous data reporting that TFAM is a key regulator of mtDNA copy number.^5, 6, 7^

To investigate the role of mtDNA copy number in the apoptosis of tumor cells, TFAM expression was downregulated by shRNA to reduce the mtDNA copy number in tumor cells. The mRNA and protein levels of TFAM in HEp-2 cells decreased significantly after transfection with shRNA-TFAM plasmids and were lowest at 24 h after transfection (Figures 2b and c). qPCR results showed that the mtDNA copy number also decreased significantly after transfection with shRNA-TFAM and also reached its lowest value at 24 h (Figure 2d). In addition, we also found that the mitochondrial mass of HEp-2 cells had increased at 24 h after the transfection with shRNA-TFAM (Figure 2e; *t*=2.836, *P*<0.05), which was confirmed in immunofluorescence detection using anti-COX IV (Supplementary Figures S2A and B), indicating a decrease in the average mtDNA copy number per mitochondria.

Next, we investigated the effect of low mtDNA copy number on the apoptosis of HEp-2 cells. In the absence of DDP, neither the HEp-2 cells with low mtDNA copy number nor the cells with normal copy number showed any obvious apoptosis (Figure 2k). In another experiment, DDP was added to the culture medium when the mtDNA copy number had reached its lowest point in the cells transfected with shRNA-TFAM. After 12 h DDP treatment, the HEp-2 cells transfected with shRNA-TFAM still had a lower mtDNA copy number than the control group even though the mtDNA copy number increased in both the groups (Figure 2j). Apoptotic cells could be found in both the groups after DDP treatment for 12 h, but the rate of apoptosis of HEp-2 cells with lower mtDNA copy number was higher than that of the control group (Figures 2f and g). This finding was further confirmed by flow cytometry (Figure 2k) (*t*=5.456, *P*<0.01). The culture medium containing DDP was then changed to fresh medium and the observations were continued until 48 h. We found significantly more apoptotic HEp-2 cells among those with lower mtDNA copy number compared with the controls both under the microscope and by flow cytometry (Figures 2h, i and k; *t*=5.600, *P*<0.01). We also observed this phenomenon in HEp-2 cells after DOX treatment for 24 h (Figure 2l; *t*=5.860, *P*<0.01). These results suggested that reduced mtDNA copy number significantly increased the sensitivity of tumor cells to chemotherapeutics. This finding was further confirmed in HNE2 and A549 cells. We found that the mRNA level of TFAM in apoptotic HNE2 cells increased, that TFAM mRNA expression was downregulated by transfection with shRNA-TFAM, and that the mtDNA copy number decreased and the apoptosis rate increased after DDP treatment (Supplementary Figures S2C–E). The same results were seen in A549 cells (Supplementary Figures S2F–H).

### EtBr blocks tumor cell proliferation and increases their sensitivity to chemotherapeutics by downregulating mtDNA copy number

**Figure 3:**
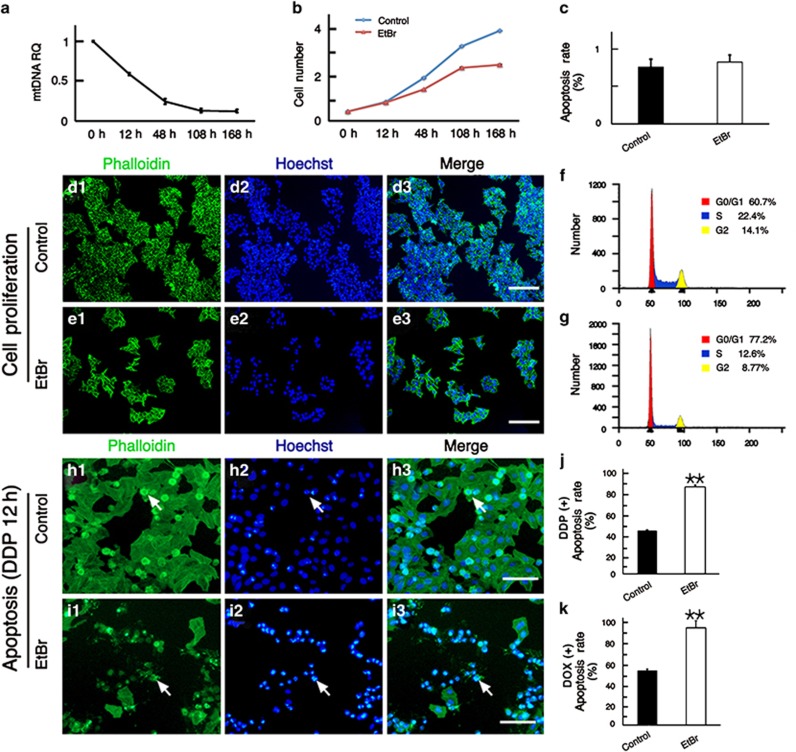
EtBr-induced downregulation of mtDNA copy number blocked the proliferation of HEp-2 cells and sensitized the cells to chemotherapeutics. (**a**) After EtBr treatment, the cells' DNA was extracted at different times for qPCR analysis. The mtDNA copy number of HEp-2 cells significantly decreased (the value was set to 1 at 0 h). (**b**) Cell numbers were detected at different times, and the proliferation of EtBr-treated cells was decreased. (**c**) Flow cytometry data showed no difference in apoptosis rate (labeling with Annexin V/PI) between the two groups at 108 h. (**d**–**e**) The cell density of EtBr-treated cells was significantly lower than the controls at 108 h (phalloidin was used to show the outlines of the cells). (**f**–**g**) The distribution of cells at different stages of the cell cycle in EtBr-treated cells and controls at 108 h. The S/G2 proportion in EtBr-treated cells was significantly smaller than in control cells. (**h**–**i**) After 12 h of DDP treatment, EtBr-treated samples contained significantly more apoptotic cells than controls. (**j**) Consistent with the results in **h**–**i**, flow cytometry data showed the apoptosis rate of EtBr-treated cells increased significantly after DDP treatment (labeling with Annexin V/PI). (**k**) Flow cytometry data at 108 h showed that the apoptosis rate of EtBr-treated cells also increased significantly after DOX treatment for 12 h (labeling with Annexin V/PI). Scale bars=50 *μ*m (**d**–**e**) and 20 *μ*m (**h**–**i**), **P<0.01

EtBr is a cationic, lipophilic DNA intercalating agent that can specifically reduce mtDNA copy number.^15, 16^ The mtDNA copy number of HEp-2 cells had decreased by almost half at 12 h after EtBr treatment and by almost 90% at 108 h (Figure 3a). Meanwhile, cell proliferation assay results showed that the cells with low mtDNA copy number proliferated slowly (Figure 3b) and had a lower cell density than the control group at 108 h even though the two groups had the same initial seeding density (Figures 3d and e). However, the two groups had no difference in apoptosis rate at 108 h (Figure 3c). Moreover, cell cycle analysis showed that the S/G2 proportion in EtBr-treated cells was significantly smaller than in the control cells (Figures 3f and g).

After EtBr treatment for 108 h followed by DDP treatment for 12 h, an increase in apoptotic cells could be seen both by microscopic observation (Figures 3h and i) and flow cytometric analysis (Figure 3j; *t*=20.398, *P*<0.01). The same increase in apoptosis was seen when HEp-2 cells were treated with DOX for 24 h after the same EtBr treatment (Figure 3k; *t*=5.640, *P*<0.01). This suggested that the sensitivity of tumor cells to chemotherapeutics increased after the downregulation of mtDNA copy number by EtBr treatment.

This finding was verified in HNE2 and A549 cells. The mtDNA copy number of EtBr-treated HNE2 cells decreased by ~80% at 108 h (Supplementary Figure S3A). DDP was then added to the medium and the cells were cultured for another 24 h. The results of flow cytometry showed that the apoptosis rate in cells with low mtDNA copy number was significantly higher compared with the control group (Supplementary Figure S3B). The same results were obtained in A549 cells (Supplementary Figures S3C and D).

We also used A549/DDP cells, which were cultured in 0.01 mM DDP medium and grew normally, to explore the relationship between mtDNA copy number and apoptosis. We found that these cells could not proliferate normally in the medium after EtBr was added, and their apoptosis rate increased gradually (Supplementary Figure S3E).

All of the above results demonstrated that tumor cells are more vulnerable to anti-tumor drugs such as DDP and DOX when the mtDNA copy number has been reduced. This suggests that chemotherapeutic-induced increases in mtDNA copy number might serve as a self-protection system for the tumor cells.

### Reduced mtDNA copy number inhibits antioxidant gene expression and raises intracellular ROS levels and sensitizes tumor cells to chemotherapeutics

**Figure 4:**
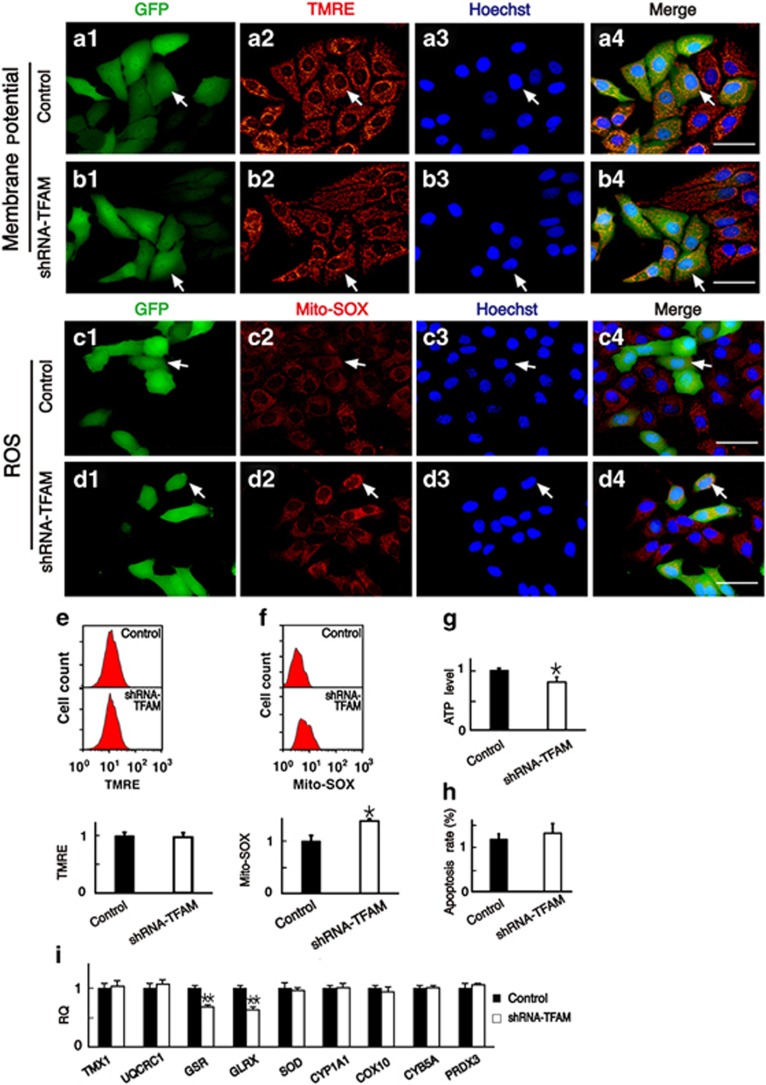
The changes in mitochondrial function in HEp-2 cells with shRNA-TFAM transfection-induced downregulation of mtDNA copy number. (**a** and **b**) HEp-2 cells were labeled with TMRE after plasmid transfection at 24 h, and the results showed that the mitochondrial membrane potential of cells transfected with shRNA-TFAM was at the same level as the controls. (**c** and **d**) We labeled the cells with Mito-SOX and found that the ROS levels of cells transfected with shRNA-TFAM were significantly higher than the controls. (**e**) The quantitative analysis of mitochondrial membrane potential by flow cytometry. (**f**) The quantitative analysis of ROS by flow cytometry. ROS levels increased in cells with shRNA-TFAM transfection. (**g**) The ATP levels in cells transfected with shRNA-TFAM were lower than the controls. (**h**) The apoptosis rate was determined by flow cytometry (labeling with DAPI/PI), and there was no difference between the two groups. (**i**) The mRNA levels of nine genes related to oxidation-reduction reactions were analyzed by qPCR, among which GSR and GLRX were significantly decreased. For all experiments, the values for the normal controls were set to 1. Scale bars=20 *μ*m, *P<0.05, **P<0.01

We measured the mitochondrial membrane potential, ROS production, and ATP levels to explore the mechanism behind the increased sensitivity of tumor cells to chemotherapeutics after the decrease of mtDNA copy number. TMRE was used for mitochondrial membrane potential analysis and Mito-SOX Red—which produces red fluorescence when it is oxidized by mitochondrial superoxide—was used to detect ROS production in mitochondria. ATP levels were measured with a luciferase-luciferin kit. At 24 h after transfection with shRNA-TFAM plasmids, immunofluorescence and flow cytometry analysis showed no change in mitochondrial membrane potential (Figures 4a, b and e), but we observed increased levels of ROS (Figures 4c, d and f; *t*=3.022, *P*<0.05) in HEp-2 cells with low mtDNA copy number. ATP levels were significantly decreased in HEp-2 cells with low mtDNA copy number (Figure 4g). Although mitochondrial dysfunction occurred, the cells with low mtDNA copy number showed no difference in the apoptosis rate compared with the control cells (Figure 4h). After shRNA-TFAM plasmid transfection in HNE2 cells, there were no significant differences in mitochondrial membrane potential or ATP levels between shRNA-TFAM and control HNE2 cells (Supplementary Figures S4A and C), however, ROS levels were still significantly increased (Supplementary Figure S4B), and the ROS levels in A549 cells after shRNA-TFAM transfection also increased (Supplementary Figure S4D).

**Figure 5:**
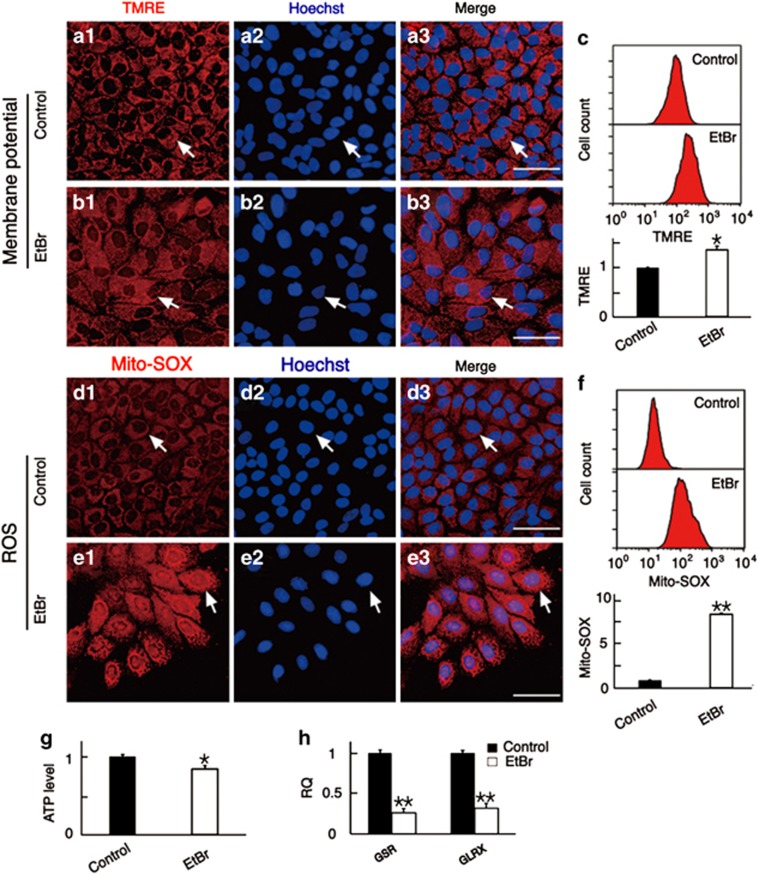
The changes in mitochondrial function in HEp-2 cells with EtBr-induced downregulation of mtDNA copy number. (**a** and **b**) HEp-2 cells were labeled with TMRE after EtBr treatment for 108 h, and the results showed that the mitochondrial membrane potentials of the cells were higher than the controls. (**c**) Flow cytometry data confirmed the results in **a** and **b**. (**d** and **e**) HEp-2 cells were labeled with Mito-SOX at 108 h, and the ROS levels in EtBr-treated cells were significantly higher than in the controls. (**f**) Flow cytometry data confirmed the results in **d** and **e**. (**g**) After EtBr treatment for 108 h, the ATP levels were lower than the controls. (**h**) The mRNA levels of GSR and GLRX were analyzed by qPCR and were found to be significantly decreased in EtBr-treated cells. For all experiments, the values for the normal controls were set to 1. Scale bars=20 *μ*m, *P<0.05, **P<0.01

The mitochondrial function of EtBr-treated HEp-2 cells was also measured to further verify the findings above. The variations of mitochondrial membrane potential and ATP levels in the EtBr-treated cells at 108 h were not entirely consistent with the results above. The mitochondrial membrane potential increased (Figures 5a, b and c), perhaps due to the increase in mitochondrial mass (Supplementary Figures S5A and B), and the ATP level was slightly decreased (Figure 5g). However, the largest difference was the increase of ROS levels in the EtBr-treated HEp-2 cells as demonstrated by immunofluorescence and flow cytometry results (Figures 5d, e and f; *t*=54.548, *P*<0.01). This was also the case for EtBr-treated HNE2 and A549 cells in which the elevated ROS levels were very obvious (Supplementary Figures S5C and D). These results further confirmed that elevated ROS levels are a common pathway after the downregulation of mtDNA copy number.

To explore the possible reasons for elevated ROS level, we measured the expression levels of nine redox-related genes by qPCR. The mRNA level of GSR and GLRX, two important antioxidant genes in the nuclear DNA, were significantly decreased in HEp-2 cells after shRNA-TFAM transfection (Figure 4i; *t*=5.100 for GSR and *t*=5.096 for GLRX, *P*<0.01) or EtBr treatment (Figure 5h; *t*=11.291 for GSR and *t*=9.767 for GLRX, *P*<0.01).

**Figure 6:**
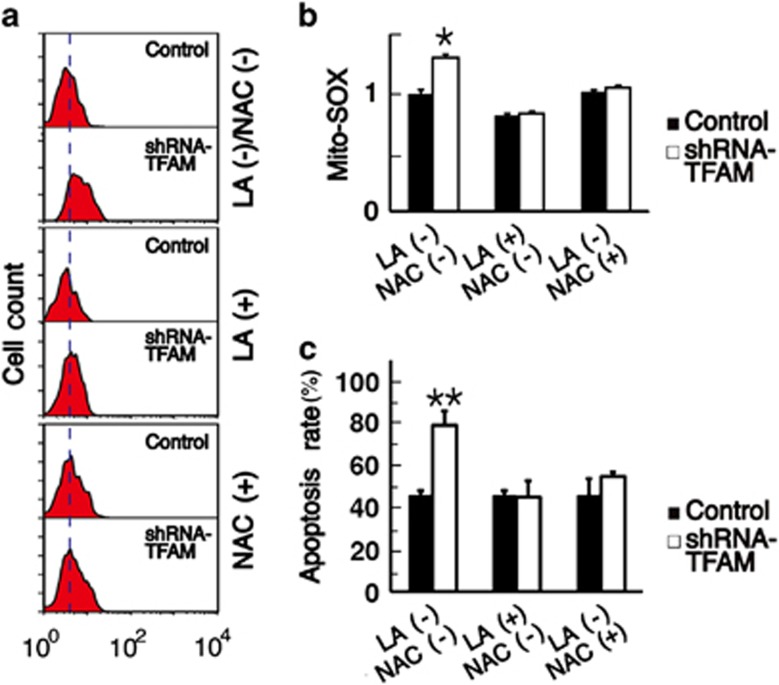
Influence of antioxidants on the apoptosis of HEp-2 cells. (**a**) Flow cytometry data showed the ROS level of cells transfected with shRNA-TFAM increased and that the increase was blocked by LA or NAC pretreatment (a vertical dotted line was added to help visualize the results). (**b**) The bar diagram of the flow cytometry data in A (the value for the first control was set to 1). (**c**) Flow cytometry data after labeling with DAPI/PI showed that when the ROS levels of cells transfected with shRNA-TFAM decreased the apoptosis rate also decreased and was the same as the control cells. *P<0.05, **P<0.01

To further confirm that ROS was the pathway through that decreased mtDNA copy number sensitizes tumor cells to chemotherapeutics, we used two ROS scavengers, LA and NAC. The ROS level increased in HEp-2 cells after shRNA-TFAM transfection for 24 h, and decreased to the same level with control cells after we pretreated the cells with 0.5 mM LA or 2 mM NAC for 12 h (Figures 6a and b). When we added DDP to the medium for 12 h, the difference in apoptosis rate between the two groups of cells disappeared (Figure 6c). We repeated the same tests in HNE2 cells and found that the ROS level also decreased to the same level as the control cells after 0.5 mM LA or 2 mM NAC pretreatment for 12 h. The apoptosis rate also decreased significantly in HNE2 cells transfected with shRNA-TFAM (Supplementary Figures S6A and B).

Together, these data support the idea that the inhibition of mtDNA copy number leads to the decreased expression level of antioxidant genes, which in turn leads to a significant increase in ROS levels that sensitizes tumor cells to chemotherapeutics.

## Discussion

The important role that mitochondria have in apoptosis has been demonstrated in previous reports.^17, 18, 19^ Many important events during apoptosis are closely related to mitochondria, including the loss of mitochondrial membrane potential, the release of cytochrome C and apoptosis-inducing factor, and changes in the activity of Bcl-2 family proteins.^17, 18, 19^ Different signal transduction pathways interact with the mitochondria to influence apoptosis, but the role of mtDNA copy number in apoptosis has remained largely unexplored.

In this study, we found that the mtDNA copy number in tumor cells increased dramatically when the cells were treated with DDP or DOX (Figure 1). However, nuclear DNA degrades at late stages in apoptotic cells, but mtDNA does not,^20^ and this might lead to an overestimation of the mtDNA copy number when the late apoptotic cells are included in qPCR analysis. Thus we sorted the early apoptotic cells by flow cytometry and found that their mtDNA copy number was also significantly increased compared with the controls (Figure 1). However, we did not observe this phenomenon in HSF cells, and the increase of mtDNA copy number in apoptotic HSF cells was not as obvious (Figure 1). These findings were confirmed by *in situ* hybridization of mtDNA (Figure 1). This change of mtDNA copy number in apoptotic cells has not previously been reported. This increase could be a cellular stress response to external factors or it could be a defensive response in tumor cells, but the mechanism involved in the relationship between increased mtDNA copy number and apoptosis remains unclear.

Mizumachi *et al.*,^21^ found that the mtDNA copy number increased in drug-resistant tumor cells of the head and neck, and the increase in mtDNA copy number reduced the production of intracellular ROS. As previously reported, elevated intracellular ROS stimulates more ROS production in a positive feedback cycle, and this can ultimately induce cell apoptosis.^22^ Therefore, an increased mtDNA copy number might be a defense mechanism of tumor cells against apoptosis, and this might be the same case in our experiment.

To test this hypothesis, we manipulated the mtDNA copy number using biological and chemical methods. Several genes have been reported to decrease the intracellular mtDNA copy number. For example, Pohjoismaki^6^ decreased the mtDNA copy number in human tumor cells through the downregulation of TFAM *in vitro*, and Guo^23^ also found that decreased TFAM activity decreased the mtDNA copy number. Other genes, like TFB2M and POLG, have been confirmed to have the same effect in regulating mtDNA copy number.^24, 25^ Consistent with these previous studies, we also found a significant increase in TFAM mRNA in apoptotic cells through gene chip analysis and qPCR, and we found that the mtDNA copy number of tumor cells decreased significantly after the downregulation of TFAM by shRNA-TFAM transfection. Also consistent with a previous report,^26^ we failed to increase the mtDNA copy number through the overexpression of TFAM *in vitro* (Supplementary Figure S7), and it has been speculated that overexpression of TFAM inhibits normal mtDNA replication, which offsets its effect on increasing mtDNA copy number.^26^ In this study, we found that decreasing the mtDNA copy number by shRNA-TFAM transfection made the tumor cells more sensitive to chemotherapeutics (Figure 2, Supplementary Figure S2).

EtBr can specifically decrease the cellular mtDNA copy number,^15, 16^ and we observed a significant decrease in the mtDNA copy number in tumor cells after EtBr treatment. EtBr can maintain the mtDNA copy number at a low level for a longer time compared with shRNA-TFAM plasmid transfection, and the use of EtBr allowed us to observe the effect of low mtDNA copy number on cell proliferation. We found that reduced mtDNA copy number decreased the growth rate and inhibited progression through the cell cycle (Figure 3). Consistent with TFAM shRNA transfection, EtBr-treated tumor cells were also more vulnerable to chemotherapeutics (Figure 3, Supplementary Figure S3).

These findings support the hypothesis that the increase of the mtDNA copy number in apoptotic cells is a self-protection mechanism in tumor cells. More importantly, these findings suggest a novel therapeutic strategy for clinical treatment of tumors by sensitizing the tumor cells to chemotherapeutic drugs by decreasing their mtDNA copy number.

In order to explore the mechanisms through which the reduced mtDNA copy number sensitized tumor cells to chemotherapeutics, we analyzed the changes in mitochondrial function (mitochondrial membrane potential, ROS production, and ATP levels) after reducing the mtDNA copy number by shRNA-TFAM transfection or by EtBr treatment. Previous studies found that decreased mtDNA copy number leads to the loss of mitochondrial membrane potential, which inhibits the proliferation of yeast cells and leads to genomic instability;^27^ that ROS can oxidize functional proteins in cells and lead to apoptosis;^28^ and that decreased ATP levels lead to elevated AMP levels that can activate the AMPK protein kinase and lead to apoptosis.^29^ However, the only consistent result we found was a significant increase in ROS after the downregulation of mtDNA copy number, and the changes in mitochondrial membrane potential and ATP level were cell specific or method specific. Thus, increased intracellular ROS appears be involved in the mechanism by which a low mtDNA copy number in tumor cells induces high sensitivity to chemotherapeutics.

Under physiological conditions, the ROS level is kept within a certain range due to the balance between production and scavenging, and this balance is the result of many mutually coordinated genes. Once this balance is altered, an increase in intracellular ROS can occur. In this study, we analyzed the mRNA levels of nine genes associated with the regulation of ROS. We found that the mRNA levels of GSR and GLRX—which are two important intracellular antioxidant genes^30, 31^—were significantly decreased (Figure 4 and 5), and this might contribute to the elevated ROS levels seen in response to decreased mtDNA copy numbers. To confirm these findings, we used two scavengers, LA and NAC, to block the ROS pathway and found that the apoptosis rate decreased significantly in tumor cells with reduced mtDNA copy number(Figure 6, Supplementary Figure S6).

In summary, this is the first report to show that the mtDNA copy number is significantly increased in apoptotic tumor cells and might serve to protect tumor cells against apoptosis. We used TFAM shRNA and EtBr to reduce the mtDNA copy number and found that this led to the increased sensitivity of tumor cells to chemotherapeutic drugs. We then demonstrated that reduced mtDNA copy number significantly increased the ROS levels in tumor cells. We hypothesize that the increase in ROS results from the decreased expression of antioxidant genes and that this makes the tumor cells more sensitive to chemotherapeutics. Finally, we blocked the ROS pathway and confirmed that ROS is the signal pathway that sensitized tumor cells to chemotherapeutics. These findings provide a potential novel therapeutic strategy for the clinical treatment of tumor cells.

## Materials and Methods

### Cell cultures and the induction of apoptosis

HEp-2 cells (obtained from the Type Culture Collection of the Chinese Academy of Sciences, Shanghai, China), HNE2 cells (human nasopharyngeal cancer cells, purchased from Yingrun Biotechnologies Inc., Changsha, China), A549 and DDP-resistant A549 (A549/DDP) cells (purchased from the cell bank of Hsiang-Ya Medical College, Changsha, China), and human skin fibroblast cell line (HSF) cells (normal adult human skin fibroblasts, purchased from Kunming cell bank of the Chinese Academy of Sciences, Kunming, China) were all grown in DMEM medium supplemented with 10% FBS, 100 IU/ml penicillin, and 100 *μ*g/ml streptomycin (Gibco Life Technologies, Gaithersburg, MD, USA). DDP was added at a final concentration of 0.01 mM to the medium for A549/DDP cells. The cells were grown at 37 °C with 5% CO_2_ and subcultured at 80% confluence using 0.25% trypsin/EDTA (Life Technologies). To induce apoptosis, DDP (DDP, Sigma, St Louis, MO, USA) was added at a final concentration of 0.12 mM, and DOX, Sigma was used at a final concentration of 0.06 mM.

### Measurement of mtDNA copy number and real-time PCR (qPCR)

Total RNA and DNA were extracted with the DNA/RNA Isolation Kit (Qiagen, Dusseldorf, Germany). Total RNA was extracted with Trizol reagent (Life Technologies) according to the manufacturer's instructions.

Reverse transcription was performed using PrimeScript RT reagent Kit with gDNA Eraser (TaKaRa, Otsu, Japan) according to the manufacturer's protocols.

For mtDNA copy number measurement, we followed the procedure as previously described.^12, 13, 14^ Three genes (ND1, COI, and COII) were used to represent mtDNA copy number (in some cases, only ND1 was used to avoid redundancy). The relative mtDNA copy number was defined as the total amount of mtDNA divided by the total amount of nuclear DNA.

We performed qPCR on an Applied Biosystems 7500 real-time PCR System (Applied Biosystems, Foster City, CA, USA) using GoTaq qPCR Master Mix (Promega, Madison, WI, USA). Validated primers were designed for each target mRNA or DNA (Supplementary Table S1). qPCR conditions were an initial denaturing step of 30 s at 95 °C followed by 40 cycles of 5 s denaturation at 95 °C, 20 s annealing at 60 °C, and 20 s extension at 72 °C. The mRNA expression values were normalized to the mRNA expression of ACTB and GAPDH. The results were calculated using the comparative cycle threshold (ΔΔCt) method.

### Plasmid transfections in cells

Human shRNA-TFAM plasmids (Hanbio, Shanghai, China) consisted of three target-specific lentiviral vector plasmids each encoding shRNAs 19–25 nucleotides in length (plus hairpin) designed to knockdown gene expression (the plasmids express GFP). The control plasmid encoded a scrambled shRNA sequence that would not lead to the specific degradation of any known cellular mRNA (the control plasmid also expresses GFP). HEp-2 or HNE2 cells were cultivated to 80% confluence in culture medium then trypsinized and washed with phosphate-buffered solution (PBS). The cells were collected in a 0.4 -cm sterile electroporation cuvette with DMEM containing no antibiotics and were transfected with a Gene Pulser Xcell Eukaryotic System (Bio-Rad, Hercules, CA, USA) at 250 V and a capacitance of 950 *μ*F.

### Preparation of the mtDNA probe and fluorescence in situ hybridization (FISH)

The probe was prepared using the FISH Tag DNA Kit (Life Technologies), and the spectrum of the intended probe was from 15 916 to 486 bases (NCBI Reference Sequence: NC_012920) in human mtDNA. The probe was amplified by PCR, and the labeling procedure consisted of two steps. In the first step, nick translation was used to enzymatically incorporate an amine-modified nucleotide into the probe template. In the second step, labeling of the purified amine-modified DNA was achieved by incubation with amine-reactive dye (see the manufacturer's instructions for detailed information).

HEp-2 or HSF cells were cultured on slides until 80% confluence, and apoptosis was induced by incubating the cells with 0.12 mM DDP for 12 h. The cells were then fixed with 4% polyoxymethylene and permeabilized with 0.5% Triton X-100. Following pepsin (0.002%, 37 °C, 10 min) and RNase (200 μg/ml, 37 °C, 30 min) treatment, the cells were immersed in 50% formamide and 2 × saline sodium citrate (SSC) until hybridization. The mtDNA was denatured by incubating the slides for 5 min at 80 °C. The hybridization solution was prepared with the probe and added to the cells and incubated at 37 °C for 24 h. In the final step, the slides were washed three times in 2 × SSC at 60 °C and imaged under a fluorescent-light microscope (model Eclipse 80i; Nikon, Tokyo, Japan). Photoshop CC was used for fluorescence intensity quantification, and fluorescence was quantified in at least 100 cells per group.

### Immunofluorescence

Tetramethylrhodamine ethyl ester perchlorate (TMRE, Sigma) was used for mitochondrial membrane potential analysis, anti-COX IV antibody (Abcam, Cambridge, UK) was used to determine mitochondrial mass, and Mito-SOX Red (Life Technologies) was used for measuring reactive oxygen species (ROS). Tumor cells were grown in a Petri dish filled with DMEM culture medium. After apoptosis induction or mtDNA copy number regulation for a certain time, the culture medium was removed from the dish, and the samples were washed with PBS. Prewarmed (37 °C) solution containing TMRE or Mito-SOX was added, and the cells were incubated with the probe for 20 min. After staining, the cells were washed in prewarmed PBS and imaged under a fluorescent-light microscope (model Eclipse 80i; Nikon). For mitochondrial mass measurement, anti-COX IV (rabbit polyclonal) was added and incubated for 8 h (4 °C) at a dilution of 1 : 200 after tumor cells were cultured, fixed with 4% polyoxymethylene, and permeabilized with 0.5% Triton X-100. The cells were washed three times with PBS and incubated for 2 h (37 °C) with red or green fluorescence-conjugated goat anti-rabbit immunoglobulin G secondary antibody (Abcam). The cells were imaged under a fluorescent-light microscope (model Eclipse 80i; Nikon). For apoptosis analysis, HEp-2 cells were treated with DDP for the desired time at which point Hoechst 33342 (Life Technologies) was added for 10 min and the cells were observed under an inverted fluorescence microscope (model Eclipse Ti; Nikon).

### Flow cytometry and cell sorting

For determining mitochondrial membrane potential and analyzing ROS production, tumor cells were cultured and, if needed, pretreated with lipoic acid (LA, Sigma) or N-acetyl-L-cysteine (NAC, Sigma) to lower the ROS level, then trypsinized, collected, and resuspended in prewarmed (37 °C) solution containing TMRE or Mito-SOX Red for 10 min followed by washing with PBS and analysis by flow cytometry (FACSCanto, BD, USA). For apoptosis analysis, the cells were washed twice with cold PBS and then resuspended in 1 × binding buffer (provided in the Annexin V/propidium iodide (PI) apoptosis detection kit, BD) at a concentration of 1 × 10^6^ cells/ml. Annexin V-FITC/PI or DAPI/PI was added and gently mixed with the cells and incubated for 15 min at room temperature in the dark. The cells were analyzed by flow cytometry as soon as possible, and all tests were repeated at least three times. Apoptotic cells labeled with Annexin V-FITC or the cells with green fluorescence after plasmid transfection were also sorted by flow cytometry.

### ATP assay

After treatment (gene transfection with shRNA-TFAM or exposure to EtBr), HEp-2 or HNE2 cells were cultured in six-well plates at a concentration of 5 × 10^5^ cells/well. The cells were trypsinized and collected by centrifugation at 600 × *g* for 5 min and washed three times with PBS. The transfected cells were sorted by flow cytometry as described in the section on flow cytometry and cell sorting.

The cells were lysed with ATP-releasing buffer containing 100 mM Tris buffer (titrated to pH 7.8 with acetic acid), 2 mM EDTA, and 1% Triton X-100. A total of 5 *μ*l of the lysate was taken for protein determination and another 5 *μ*l of the lysate was added to a 96-well plate. ATP concentrations in the lysates were quantified in triplicate using an ATP determination kit (luciferase-luciferin; Life Technologies) in a BioTek FL600 microplate reader according to the manufacturer's instructions (results were adjusted according to the protein concentration).

### Isolation of mitochondria

Cells were trypsinized, collected by centrifugation at 600 × *g* for 5 min at 4 °C, washed with ice cold PBS, and resuspended in 500 *μ*l of Cytosol Extraction Buffer (BioVision, San Francisco, CA, USA). After incubation on ice for 10 min, the cells were homogenized in an ice cold dounce tissue grinder and transferred to a 1.5-ml tube and centrifuged at 700 × *g* for 10 min at 4 °C. The supernatant was transferred to a fresh 1.5 ml tube and centrifuged at 10 000 × *g* for 30 min at 4 °C. The supernatant was removed and the pellet was resuspended in 500 *μ*l cytosol extraction buffer and centrifuged again at 10 000 × *g* for 30 min at 4 °C. The remaining pellet consisted of mitochondria that were lysed for western blot or labeling with MitoTracker Green FM (Life Technologies) for the analysis of mitochondrial mass in a BioTek FL600 microplate reader.

### Western blot

The mitochondria were lysed with RIPA Lysis Buffer (Beyotime, Shanghai, China) according to the manufacturer's instructions to obtain the cytoplasmic mitochondrial proteins. Protein concentrations were measured using an Enhanced BCA Protein Assay Kit (Beyotime) according to the manufacturer's instructions using GAPDH as the reference protein. TFAM levels were monitored using a commercially available anti-TFAM rabbit polyclonal antibody (Santa Cruz Biotechnology, CA, USA), and GAPDH was measured using a mouse monoclonal antibody (Abcam). Peroxidase-conjugated goat anti-rabbit (or anti-mouse) immunoglobulin G (Abcam) was employed as the secondary antibody. The proteins were bound to polyvinylidene fluoride membranes and detected using a SuperSignal West Pico chemiluminescent substrate kit (Thermo Scientific, Waltham, MA, USA) according to the manufacturer's instructions.

### Cell proliferation and cell cycle assays

Cells were trypsinized, collected by centrifugation at 600 × *g* for 5 min, resuspended in culture medium, and plated on 96-well plates at 1,000 cells/well with four replicates. After 24 h incubation, EtBr was added at a final concentration of 0.25 μM (controls only received a similar volume of DMEM). Cell numbers were detected with the CCK-8 kit (Dojindo, Japan) at different times after incubation. For cell cycle analysis, harvested cells were washed with PBS and fixed with ice cold 70% ethanol overnight and then treated with RNase and stained with PI. After staining, cell cycle analysis was carried out using flow cytometry (FACSCanto, BD) and ModFIT LT 3.2 (Verity Software House, Greenville, SC, USA).

### Statistics

For all experiments, the values for the normal controls were set to 1. Statistical analyses were performed with SigmaPlot 12.3 (San Jose, CA, USA). A two-tailed *t*-test was used to compare the values between the groups. The difference was considered significant when *P*<0.05.

## Abbreviations

mtDNA: mitochondrial DNA
HSF: human skin fibroblast cell line
DDP: cisplatin
DOX: doxorubicin
ROS: reactive oxygen species
TFAM: Mitochondrial transcription factor A
ATP: adenosine triphosphate
HNE2 cells: human nasopharyngeal cancer cells
TMRE: Tetramethylrhodamine ethyl ester perchlorate
LA: lipoic acid
NAC: N-acetyl-L-cysteine
PI: Propidium iodide
SSC: saline sodium citrate
