# Supplementary material for: Reduced mtDNA copy number increases the sensitivity of tumor cells to chemotherapeutic drugs
Source: Cell Death Dis. 2015 Apr 2;6(4):e1710–. doi: 10.1038/cddis.2015.78 (PMC4650546; doi:10.1038/cddis.2015.78)
Supplement: Supplementary Information [file cddis201578x1.doc]

Table S1: PCR primer sequences used in the experiments.

|  | **Forward Primer** | **Reverse Primer** |
| --- | --- | --- |
| **ND1** | CCTAGCCGTTTACTCAATCCT | TGATGGCTAGGGTGACTTCAT |
| **COI** | TTCGCCGACCGTTGACTATTCTCT | AAGATTATTACAAATGCATGGGC |
| **COII** | CGTCTGAACTATCCTGCCCG | TGGTAAGGGAGGGATCGTTG |
| **ACTB(DNA)** | CATCTCTTGCTCGAAGTCCA | ATCATGTTTGAGACCTTCAACA |
| **TFB2M** | CACCTGCTATGTCTTCTCG | AAAGGGATGTCTGCTGT |
| **SSBP1** | GTCCGAAACAACTACCA | CCTTCCACCTGTCTCAA |
| **POLG** | CCTTGTGGGTGCTGATGTG | GGCAAAGTGGGCGTCTC |
| **POLG2** | AGTTTGCCATGAGTCCA | TTCCACAGGGTTTCTATT |
| **TFAM** | ATGGCGTTTCTCCGAAGCAT | CAGATGAAAACCACCTCGGTAA |
| **TMX1**  **UQCRC1** | CGCTATCAGGGTCCAA  TTTGTCTGTGACCGAATGAA | TGAAACGGGCTCAATAC  GCTGCGTCCGATGTCCT |
| **GSR** | ATGCTGATTATGGCTTTCC | CCTCTATTGTGGGCTTGG |
| **GLRX** | CAAACAAGGGCTTCTGG | TGCATCCGCCTATACAAT |
| **SOD** | GGGCATCATCAATTTCG | AGCCTGCTGTATTATCTCCA |
| **CYP1A1** | TTCATCCCTATTCTTCGCTAC | GTGCTCCTTGACCATCTTCT |
| **COX10** | AGGCTCTGTCTGGTATCTTG | GCTTGGGTCTTACTTGCTG |
| **CYB5A** | ATGGCAGAGCAGTCGG | GTTCTCAGTAGCGTCACCT |
| **PRDX3** | TGCCTGGATAAATACACC | GTCTCGGGAAATCTGCT |
| **ACTB (cDNA)** | TCAAGATCATTGCTCCTCCTGAG | ACATCTGCTGGAAGGTGGACA |
| **GAPDH** | CTGGTAAAGTGGATATTGTTGCCAT | TGGAATCATATTGGAACATGTAAACC |
| **mtDNA probe** | TCTTGTAAACCGGAGATGAA | GATGAGATTAGTAGTATGGGAGTG |

Supplementary figure legends:

Figure S1. The mtDNA copy number variation in apoptotic HNE2 and A549 cells (both were treated for 24 h by DDP or DOX) based on qPCR analysis. (A-B) The DDP- or DOX-induced early apoptotic cells and the normal cells were sorted by flow cytometry. Subsequent qPCR analysis showed that the mtDNA copy number increased in apoptotic HNE2 cells. (C-D) The tests were repeated in A549 cells, and the mtDNA copy number increased in DDP- or DOX-induced apoptotic A549 cells. **p < 0.01.

Figure S2. The mitochondrial mass change in HEp-2 cells after shRNA-TFAM transfection and the down-regulation of TFAM expression in HNE2 and A549 cells. (A-B) Anti-COX IV was used to measure the mitochondrial mass in HEp-2 cells after shRNA-TFAM transfection at 24 h, and the results showed that the mitochondrial mass increased. (C) The qPCR data showed that the mRNA level of TFAM increased in apoptotic HNE2 cells. (D) After shRNA-TFAM transfection, the mtDNA copy number of HNE2 cells decreased and reached its lowest point at 48 h. (E) At 48 h, we treated the cells with DDP for 24 h, and subsequent flow cytometry showed that the apoptosis rate of cells transfected with shRNA-TFAM increased. (F-H) We repeated the tests in A549 cells and found that the mRNA level of TFAM increased in apoptotic A549 cells, the mtDNA copy number decreased after shRNA-TFAM transfection and reached its lowest point at 24 h, and the apoptosis rate of cells with low mtDNA copy number increased after DDP treated for 24 h. Scale bars = 20 μm, *p < 0.05, **p < 0.01.

Figure S3. EtBr-induced down-regulation of mtDNA copy number sensitized HNE2, A549, and A549/DDP cells to DDP. (A) The qPCR data showed that the mtDNA copy number of HNE2 cells was significantly decreased compared to controls after EtBr treatment for 108 h. (B) Flow cytometry data showed that after 24 h DPP treatment the apoptosis rate of EtBr-treated cells was significantly higher than the controls. (C-D) The results in A549 cells were consistent with those in HNE2 cells (all the treatments were the same with HNE2 cells). The mtDNA copy number decreased and the apoptosis rate increased significantly after EtBr treatment. (E) Flow cytometry data showed that A549/DDP cells growing in the medium that contained only EtBr or 0.01mM DDP had no increased apoptosis rate even when we observed them for 10 days, but they had a gradually increased apoptosis rate when both EtBr and DDP were added in the medium. **p < 0.01.

Figure S4. The changes in mitochondrial function in HNE2 and A549 cells after shRNA-TFAM transfection-induced down-regulation of mtDNA copy number (all were detected by flow cytometry except for ATP). (A) There was no significant difference between the two groups in terms of mitochondrial membrane potential after shRNA-TFAM transfection at 48 h. (B) The ROS levels of HNE2 cells transfected with shRNA-TFAM increased significantly. (C) The ATP of the two groups was extracted and measured, and no significant difference was observed. (D) The ROS levels of A549 cells also increased significantly after shRNA-TFAM transfection at 24 h. *p < 0.05.

Figure S5. The mitochondrial mass change of EtBr-treated HEp-2 cells and the ROS level change of EtBr-treated HNE2 and A549 cells (at 108 h). (A-B) Anti-COX IV was used to measure the mitochondrial mass in EtBr-treated HEp-2 cells, and the results showed that the mitochondrial mass increased. (C-D) The ROS levels increased significantly after EtBr-induced down-regulation of mtDNA copy number in HNE2 and A549 cells (based on flow cytometry). Scale bars = 20 μm, ** p < 0.01.

Figure S6. Influence of antioxidants on the apoptosis of HNE2 cells. (A) The flow cytometry data showed that the ROS levels of cells transfected with shRNA-TFAM increased and that the increase was blocked by LA or NAC pretreatment (the value for the first control was set to 1). (C) When the ROS levels in cells transfected with shRNA-TFAM decreased, the apoptosis rate also decreased significantly. *p < 0.05, **p < 0.01.

Figure S7. The plasmid containing human TFAM cDNA was transfected into HEp-2 cells, but over-expression of TFAM failed to increase the mtDNA copy number except for a small, transient increase at 16 h. * p < 0.05.
