# Supplementary figures and images for: Reduced mtDNA copy number increases the sensitivity of tumor cells to chemotherapeutic drugs
Source: Cell Death Dis. 2015 Apr 2;6(4):e1710–. doi: 10.1038/cddis.2015.78 (PMC4650546; doi:10.1038/cddis.2015.78)

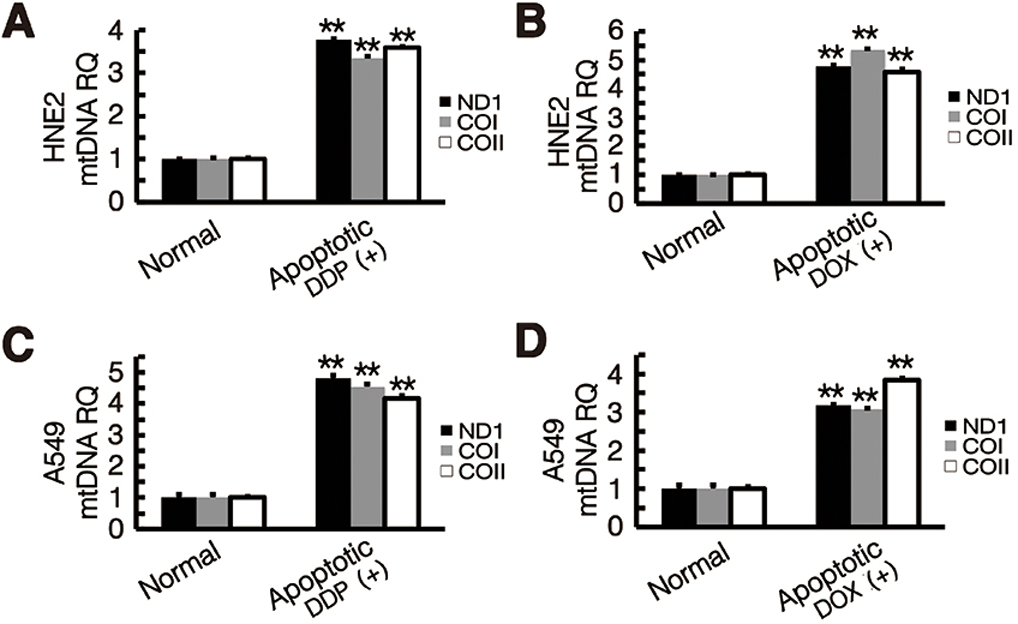

Supplement: Supplementary Figure 1 [file cddis201578x2.tif]

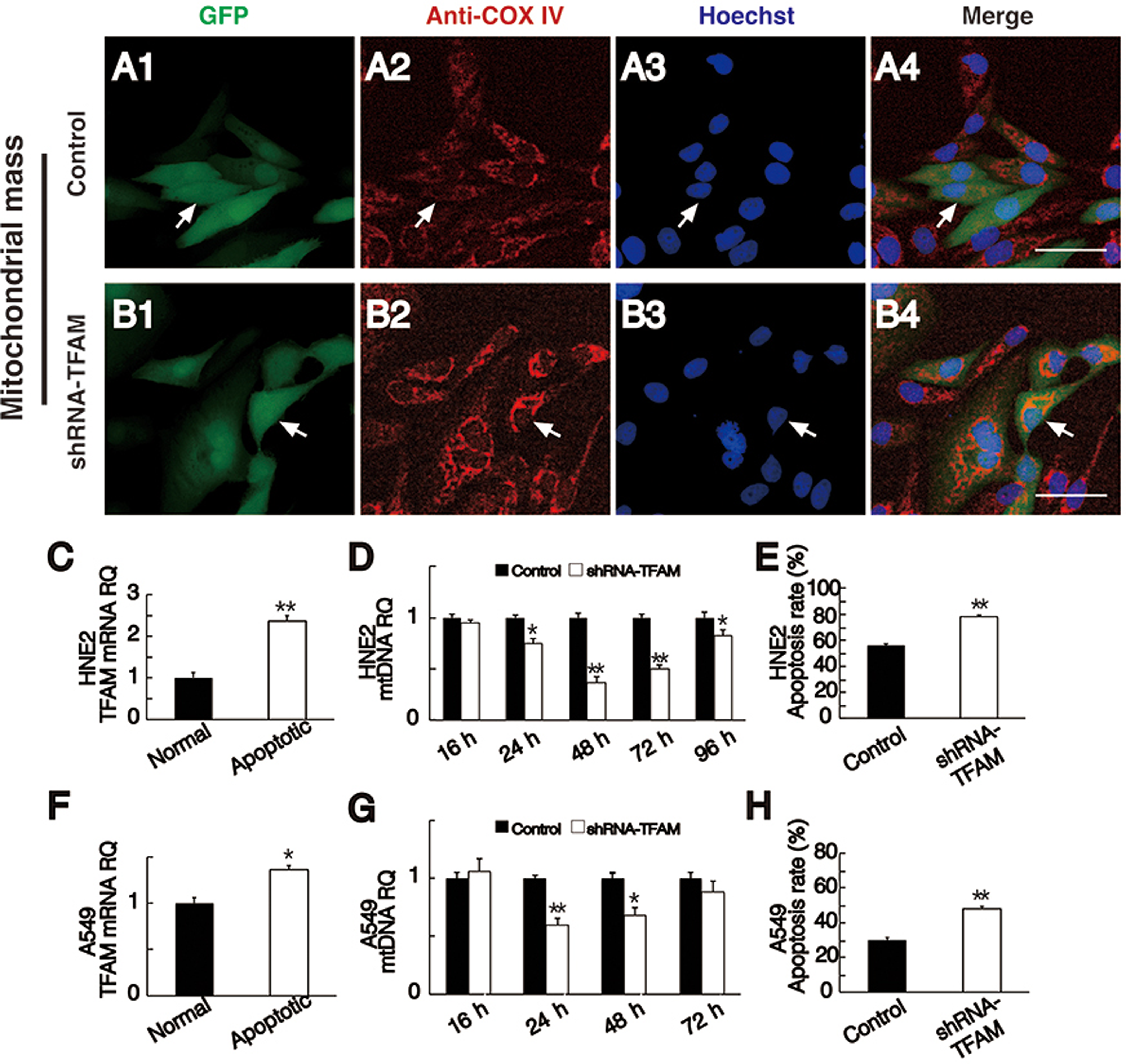

Supplement: Supplementary Figure 2 [file cddis201578x3.tif]

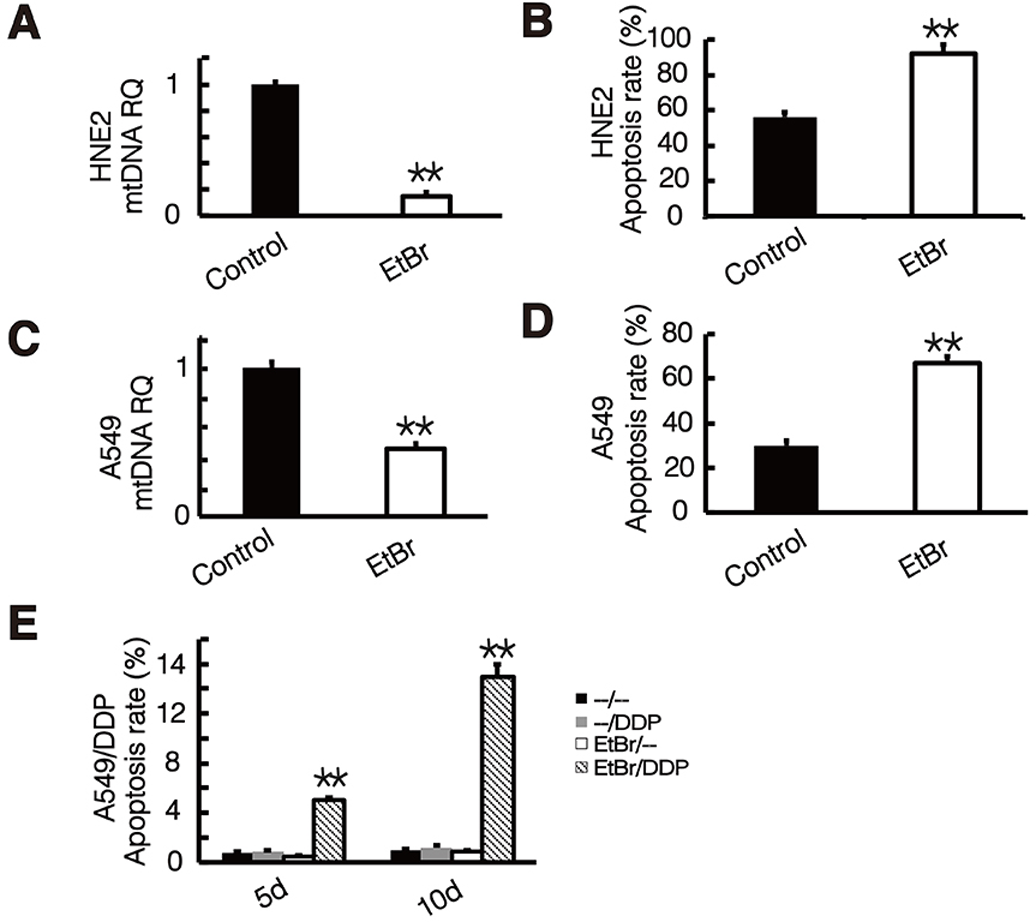

Supplement: Supplementary Figure 3 [file cddis201578x4.tif]

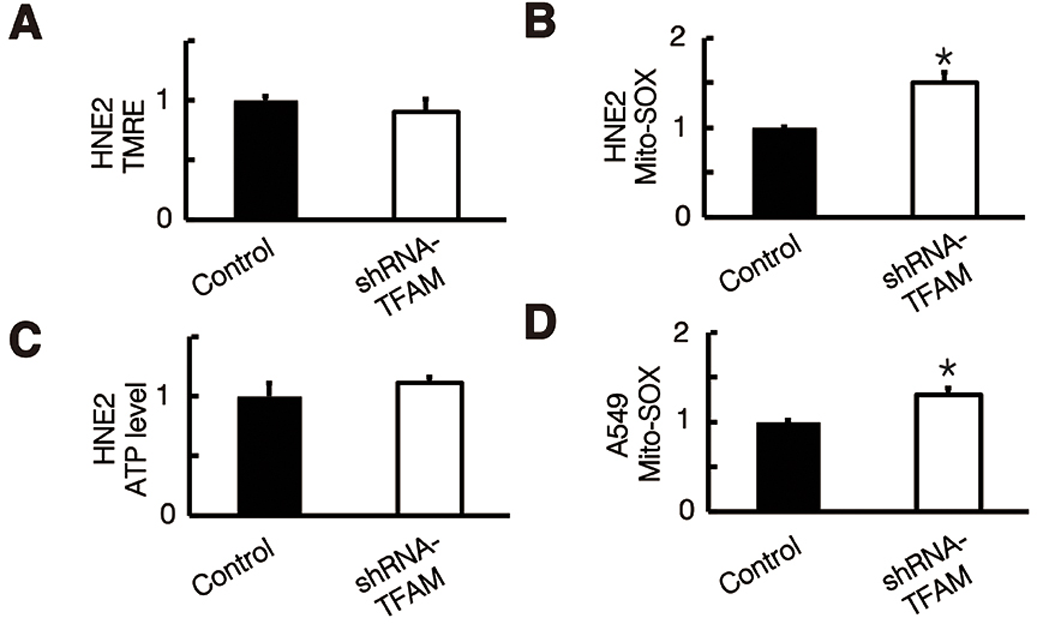

Supplement: Supplementary Figure 4 [file cddis201578x5.tif]

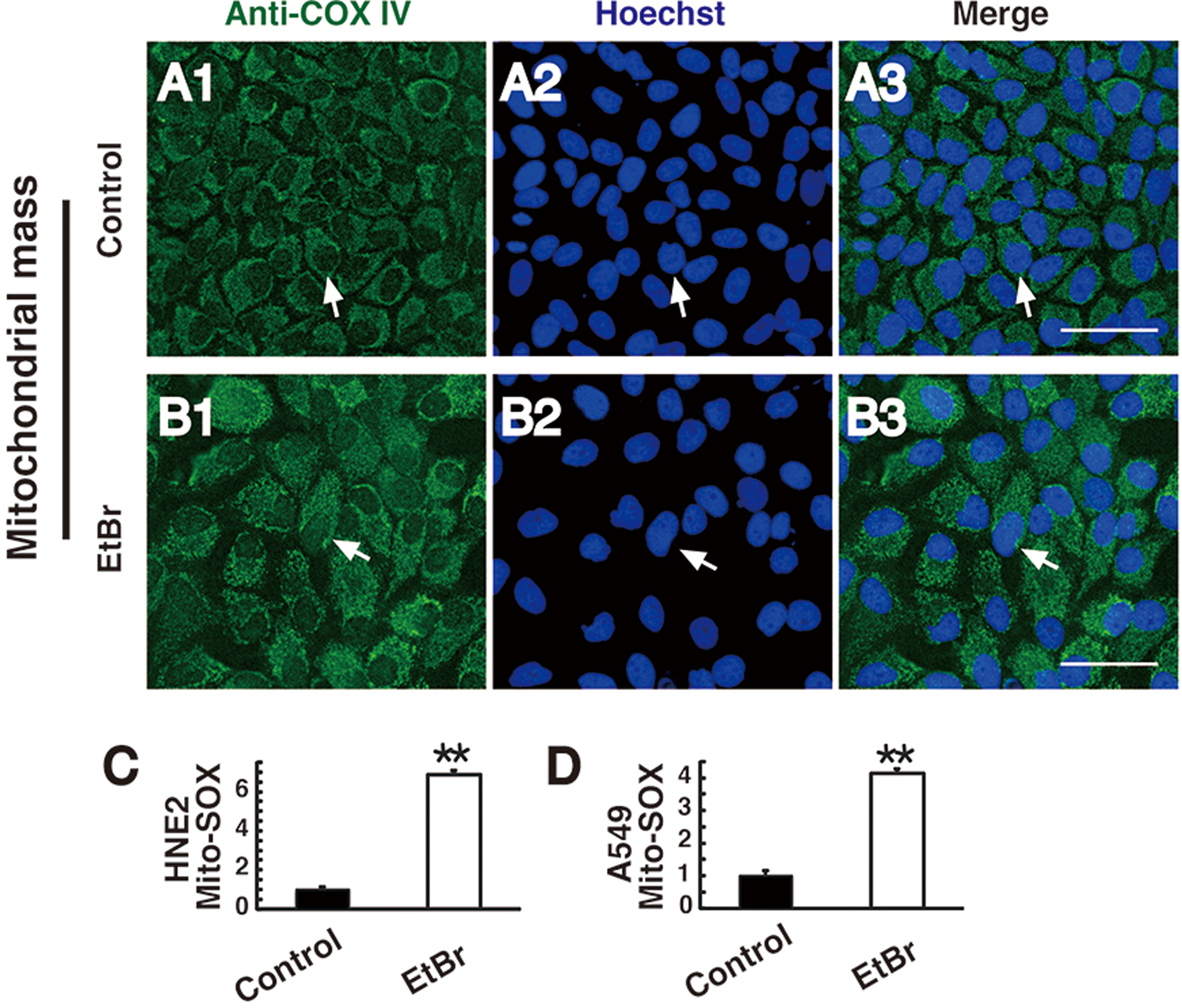

Supplement: Supplementary Figure 5 [file cddis201578x6.tif]

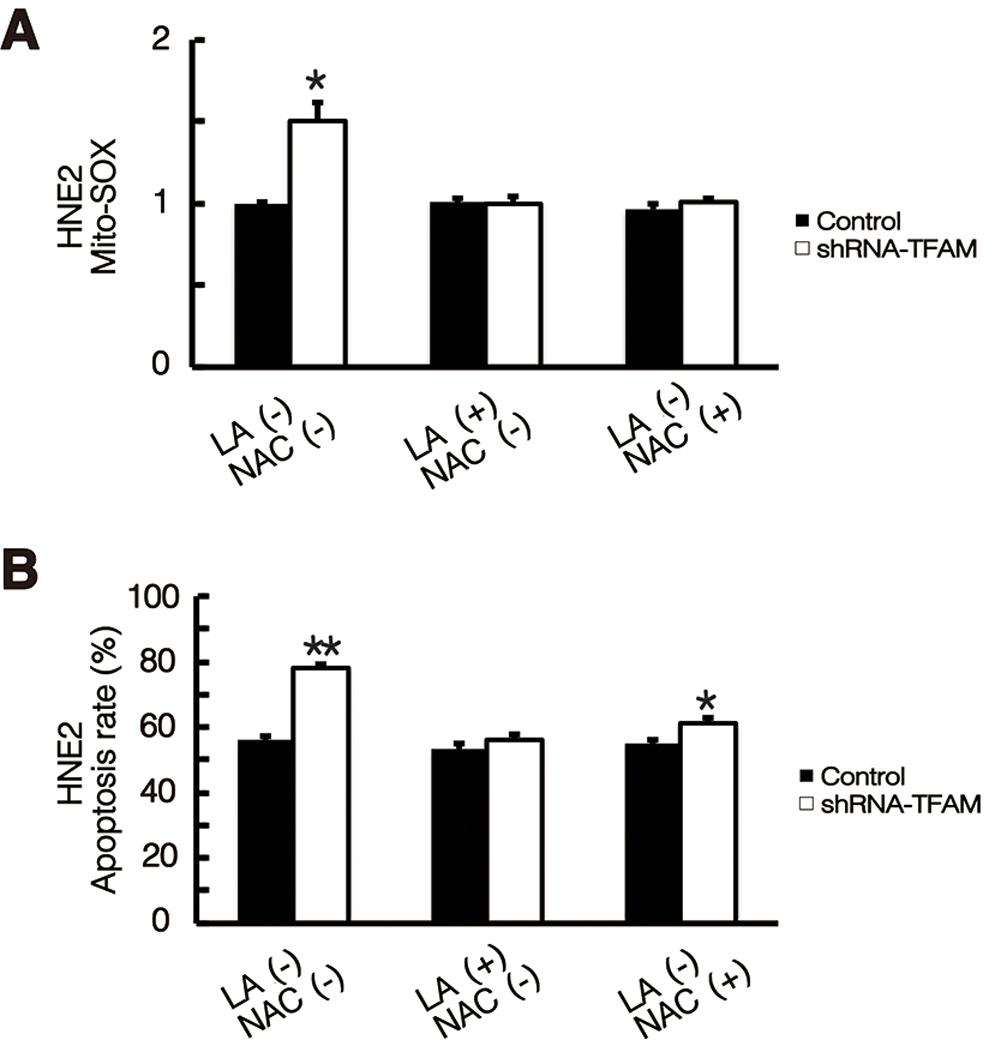

Supplement: Supplementary Figure 6 [file cddis201578x7.tif]

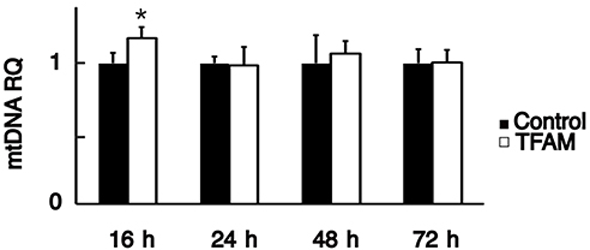

Supplement: Supplementary Figure 7 [file cddis201578x8.tif]
